# Misdiagnosed as a perianal abscess: case report of perianal endometriosis

**DOI:** 10.1093/jscr/rjae539

**Published:** 2024-08-30

**Authors:** Xuejiao Ding, Yichao Tao, Cong Hu, Xiaofeng Wu

**Affiliations:** Department of Ultrasound Imaging, Xiaogan Hospital, Affiliated to Wuhan University of Science and Technology, Xiaogan Central Hospital, Xiaogan, Hubei, PR China; Department of Ultrasound Imaging, Xiaogan Hospital, Affiliated to Wuhan University of Science and Technology, Xiaogan Central Hospital, Xiaogan, Hubei, PR China; Department of Ultrasound Imaging, Xiaogan Hospital, Affiliated to Wuhan University of Science and Technology, Xiaogan Central Hospital, Xiaogan, Hubei, PR China; Department of Ultrasound Imaging, Xiaogan Hospital, Affiliated to Wuhan University of Science and Technology, Xiaogan Central Hospital, Xiaogan, Hubei, PR China

**Keywords:** perianal endometriosis, ultrasound, perianal abscess

## Abstract

Perianal endometriosis represents a rare form of endometriosis occurring outside the pelvic cavity. Owing to its infrequency in clinical practice, this condition is highly susceptible to misdiagnosis and inappropriate treatment. This case report details a young female patient who was erroneously diagnosed with a perianal abscess. We conducted a para-anal mass resection under spinal anesthesia, and subsequent histopathological examination definitively confirmed the diagnosis of perianal endometriosis.

## Introduction

Endometriosis is a common benign condition among women of reproductive age, with a peak incidence between 24 and 29 years [1]. The growth and invasion of endometrial glands and stroma outside the endometrium frequently lead to pelvic pain, including dysmenorrhea, painful bowel movements, and dyspareunia. It can also result in infertility, menstrual irregularities, and the formation of masses [2]. Ectopic endometriosis primarily affects the ovaries, with 25% occurring in areas outside the reproductive system, such as the sigmoid colon, rectum, abdominal wall, and perineum [3]. The incidence of perianal endometriosis (PE) is only 0.3%–1% [4]. Clinically, it often presents with perianal masses, pain, and discomfort around the anus, which are often mistaken for perianal abscesses. Studies have indicated a strong correlation between this condition and the development of endometrial lesions resulting from lateral perineal incision scars [5]. This article presents a case of PE mistaken for perianal abscess to enhance the awareness of the disease and minimize the rate of misdiagnosis.

## Case report

**Figure 1:**
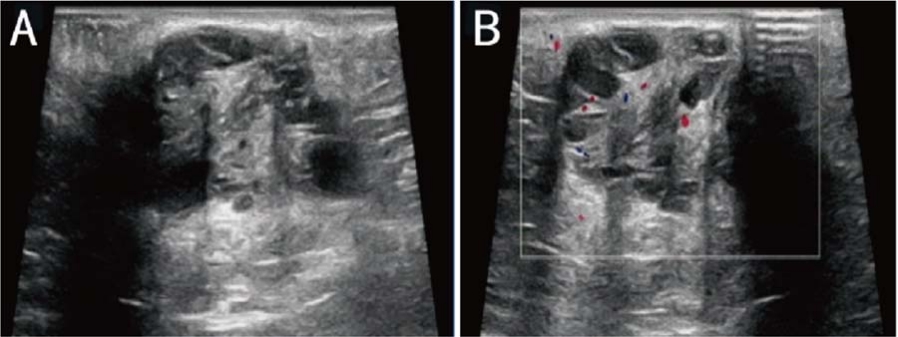
Ultrasound: (A) 2D ultrasound shows a mixed echo with a range of about 4 cm in the subcutaneous soft tissue around the left side of the anus, presenting as cystic-solid; (B) color doppler shows a small amount of punctate blood flow signal inside and around the mixed echo.

**Figure 2:**
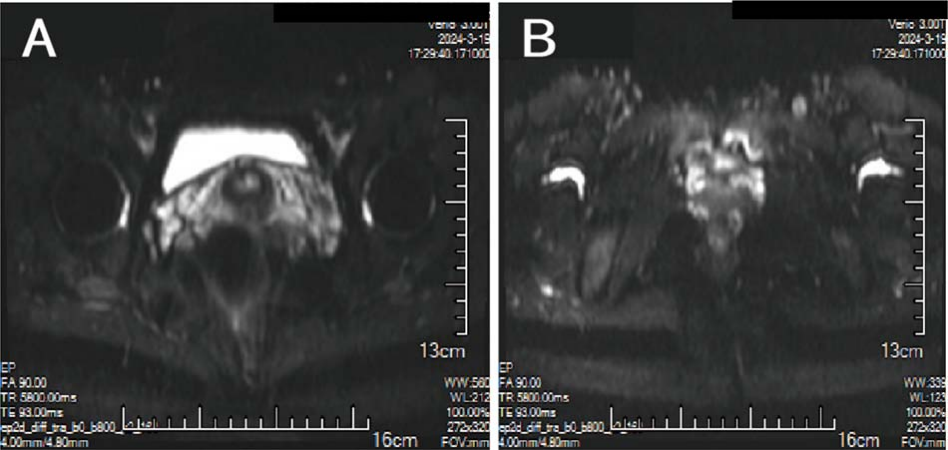
MRI: (A, B) A cluster of low/high mixed signals such as T1 and T2 can be seen on the left side of the anus, and the DWI signal is partially increased.

**Figure 3:**
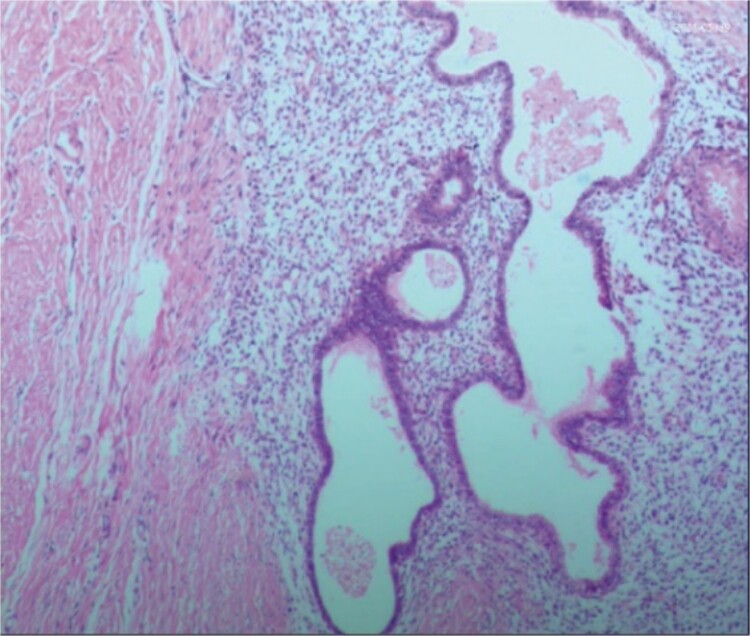
Postoperative pathology: the endometrial glands have typical stroma, blood, and macrophages containing hemosiderin.

A 38-year-old married female patient presented with a lump near her anus 10 years ago, without apparent cause or symptoms such as redness, swelling, heat, or pain, nor cough, abdominal pain, or diarrhea. No specific treatment was administered. The patient now reports that the lump has grown larger and is accompanied by increasing swelling and discomfort. Seeking further diagnosis and treatment, she visited our outpatient department, where she was admitted with the condition “perianal lump.” Physical examination revealed a body temperature of 36.5°C, pulse rate of 86 beats/min, respiratory rate of 19 beats/min, and a blood pressure of 134/85 mmHg. Admission symptoms include perianal swelling and pain, with itching; no signs of fever, chills, rupture, or pus discharge; bowel movements once or twice daily, and normal urination. The patient has one child and underwent a lateral epidural resection 12 years ago due to natural childbirth. Specialist examination revealed a 4-cm lump with a hard texture at 7–11 o’clock next to the anus, and a radial surgical incision at the 7 o’clock anal margin. Digital rectal examination found no lump, depression, or induration in the anus, and no blood on fingertips. The initial diagnosis was a perianal lump. Auxiliary examinations included ultrasound, which revealed a 4-cm lump in the subcutaneous soft tissue near the anus with minor blood flow signals within and around it, suggestive of an inflammatory lesion (Fig. 1A and B); and pelvic MRI, which suggested a left perianal lump consistent with a perianal abscess (Fig. 2A and B). Under spinal anesthesia, the perianal lump was removed, and postoperative pathology confirmed the presence of endometriosis (Fig. 3).

## Discussion

PE represents a specific type of endometriosis, with related studies reporting that iatrogenic implantation is closely linked to the development of endometriotic lesions in perineal lateral incision scars [6]. PE exhibits symptoms similar to those of perianal abscesses, with both conditions originating around the anus. These conditions are clinically rare, often leading to misdiagnosis due to a lack of understanding among clinicians. In contrast to perianal abscesses, PE typically presents as recurrent perianal masses accompanied by cyclical pain, with the masses enlarging during menstruation and subsequently shrinking or disappearing thereafter [7, 8]. Patients often have a history of perineal lacerations or episiotomy, with surgical scars typically visible on specialized examinations. Anorectal examinations reveal no signs of anal sinus infection [9]. Ultrasonography demonstrates heterogeneous hypoechoic masses with punctuate or branching blood flow signals [10]. MRI exhibits a sensitivity of 90%–92% and a specificity of 91%–98% for the diagnosis of PE. MRI not only precisely locates the lesion but also outlines its extent, enabling differentiation from various anorectal conditions, thereby greatly aiding in the disease’s diagnosis [11]. Histopathological examination serves as the definitive diagnostic method for this condition, requiring the microscopic identification of two out of three components: endometrial stromal cells, glands, and hemosiderin, to confirm the diagnosis [12].

The treatment for PE emphasizes a combined approach of surgery and medication, with surgery as the primary method and medication as a supplement. Although hormone suppression is typically the primary treatment for extrapelvic endometriosis, surgical removal is the optimal treatment for PE due to the anatomical fibrous structure of the anal sphincter. Only surgery can provide the histological specimens needed to rule out the rare development of malignant tumors in patients with extragenital endometriosis [13].

In this case, the patient was diagnosed with endometriosis through specialized examination, which revealed palpable masses of varying sizes around the anus. Postoperative pathological biopsy confirmed the diagnosis. However, in clinical practice, a small number of patients with atypical symptoms are often misdiagnosed with perianal abscess. Therefore, when there is high suspicion, a comprehensive and detailed medical history should be taken, considering the possibility of this disease, and relevant examinations should be conducted.

The misdiagnosis of this case was due to the patient not experiencing periodic pain for over a decade, with nonspecific clinical symptoms that were challenging to diagnose. Additionally, the rarity of the condition combined with a lack of familiarity among clinicians and radiologists regarding its characteristics, insufficiently detailed medical history, and a failure to perform comprehensive analysis and differential diagnosis, led to it being incorrectly diagnosed as a perianal abscess.

PE is a female-specific condition closely linked to menstruation, pregnancy, and childbirth. In clinical practice, for patients with perianal masses and a history of endometriosis, perianal surgery, or episiotomy, a thorough examination of their medical, menstrual, and reproductive history is essential. Comprehensive specialized examinations and a multidimensional approach to analysis and thinking are crucial to minimize misdiagnosis of this condition.

## Conflict of interest statement

The authors declare that there are no conflicts of interest.
